# Circulating microvesicles correlate with radiation proctitis complication after radiotherapy

**DOI:** 10.1038/s41598-022-21726-y

**Published:** 2023-02-04

**Authors:** Alexandre Ribault, Mohamed Amine Benadjaoud, Claire Squiban, Laurent Arnaud, Coralie Judicone, Aurélie S. Leroyer, Alexandra Rousseau, Christelle Huet, Chandan Guha, Marc Benderitter, Romaric Lacroix, Stephane Flamant, Emily I. Chen, Jean-Marc Simon, Radia Tamarat

**Affiliations:** 1 Institut de Radioprotection Et de Sûreté Nucléaire (IRSN), PSE-SANTE, BP17, 92262 Fontenay-aux-Roses Cedex, France; 2 Department of Hematology, Biogenopole, CHU La Timone, APHM, Marseille, France; 3 Aix-Marseille University, C2VN, INSERM 1263, INRAE 1260, Marseille, France; 4 Hôpital Saint Antoine, APHP, Unité de Recherche Clinique de L’Est Parisien (URC-Est), Paris, France; 5 Department of Radiation Oncology, Albert Einstein College of Medicine, New York, USA; 6 Department of Pharmacology, Columbia University Medical Center, New York, USA; 7 Hôpital Pitié-Salpêtrière, Sorbonne Université́, Service d’Oncologie Radiothérapie, Assistance Publique-Hôpitaux de Paris (APHP), 75013 Paris, France

**Keywords:** Biomarkers, Radiotherapy

## Abstract

In a large retrospective study, we assessed the putative use of circulating microvesicles (MVs), as innovative biomarkers of radiation toxicity in a cohort of 208 patients with prostate adenocarcinoma overexposed to radiation. The level of platelet (P)-, monocyte (M)- and endothelial (E)-derived MVs were assessed by flow cytometry. Rectal bleeding toxicity scores were collected at the time of blood sampling and during the routine follow-up and were tested for association with MVs using a multivariate logistic regression. MVs dosimetric correlation was investigated using dose volume histograms information available for a subset of 36 patients. The number of PMVs was significantly increased in patients with highest toxicity grades compared to lower grades. Risk prediction analysis revealed that increased numbers of PMVs, and an increased amount of MMVs relative to EMVs, were associated with worst rectal bleeding grade compared to the time of blood sampling. Moreover, a significant correlation was found between PMV and MMV numbers, with the range of doses up to the median exposure (40 Gy) of bladder/rectum and anterior rectal wall, respectively. MVs could be considered as new biomarkers to improve the identification of patients with high toxicity grade and may be instrumental for the prognosis of radiation therapy complications.

## Introduction

In the context of radiotherapy, exposition of normal tissues surrounding tumors to ionizing radiations may result in serious complications^1^. Hence, Pelvic radiation disease occurs in more than 90% of patients after abdominopelvic radiotherapy, and is characterized by frequent bowel movements and painful rectal bleedings (RB)^2,3^. Acute radiation-induced toxicity and inflammatory processes of the gastrointestinal tract seem to be linked to the risk of developing late toxicity, termed “consequential late effects”^4^. Preclinical studies have shown that radiation toxicity leads to severe chronic mucosal ulceration resulting from inflammatory processes, vascular damage and necrosis^5–7^. However, the severity of gastrointestinal damages during radiotherapy remains unpredictable despite the studies of several potential biomarkers^2^. Therefore the identification of new biological markers may constitute an important predictive tool to stratify patients with radiation-induced toxicity of abdomino-pelvic radiotherapy.

Cell-derived extracellular vesicles (EVs), including microvesicles (MVs) and exosomes, contain various biological molecules such as nucleic acids, proteins and structural molecules, the nature of which is highly dependent on the parental cell and the stimulus applied on^8^. MVs originate from the cellular membrane of healthy, activated and injured cells, with a size range between 100 and 1000 nm in diameter. Exosomes are smaller EVs (between 40 and 100 nm in diameter) and are released in the extracellular space after fusion of intracellular multi-vesicular bodies with the plasma membrane^8^. Importantly, EVs are considered as vectors of biological information which participate to intercellular communication, as they can reach neighboring or distant cells to deliver their content.

Recent evidence suggests that EVs may not be just a consequence of disease, but also actors of pathological processes and could therefore serve as markers and mediators of pathologies such as cardiovascular diseases^9^. MVs have been studied as potential diagnostic and prognostic biomarkers in cardiovascular and inflammatory diseases, as well as in cancer, endothelial injury or high-grade carotid stenosis^10–14^. More specifically, MVs have been shown to exert a procoagulant^15,16^ or an anti-coagulant activity^17,18^ in hemostatic and thrombotic disorders. MV-induced coagulation relies, at least in part, on their ability to expose phosphatidylserine and tissue factor (TF) on the external side of their membrane^19^. As such, EVs are involved in various biological processes such as hemostasis, inflammation, and endothelium activation^12,20,21^.

In the present study, we analyzed blood samples harvested from a cohort of 208 patients who were overexposed to radiation during radiotherapy for prostate adenocarcinoma^22^. We investigated whether EVs (MVs and exosomes) isolated from peripheral blood could be used as potential biomarkers in association with the severity grade depicting the radiotherapy complications in this cohort. We also examined whether combining EV protein profiling with pathological features could correlate to severity grades of rectal complications.

## Materials and Methods

### Study design

**Figure 1:**
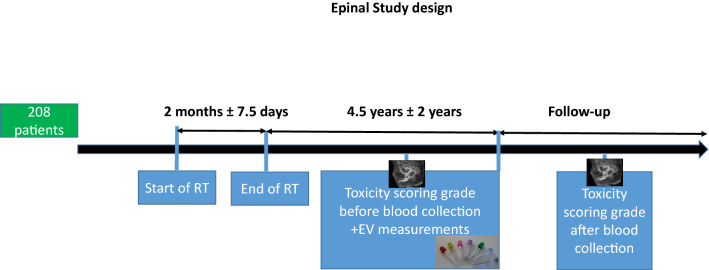
Main steps of the EPINAL study protocol. Blood samples were collected for circulating biomarkers analysis in the years following the end of the RT; toxicity scoring was performed at the time of blood collection and during follow-up.

This investigation was performed after approval by a local Human Investigations Committee. This study was approved by the “Comité de Protection des Personnes” (ethical committee) and the “Commission Nationale de l’Informatique et des Libertés” (CNIL) according to French regulations, and all methods were performed in accordance with the ethical principles as contained in the Declaration of Helsinki. Written informed consent was obtained from all subjects and/or their legal guardian(s). https://clinicaltrials.gov/ registered number: NCT00773656 (Fig. 1). Following an over-irradiation during their treatment against a prostate adenocarcinoma, patients from Epinal general hospital (France) developed a chronic radiation enteritis with RB with distinct severity degrees and constituted the EPOPA (Epinal Patients Overexposed for a Prostate Adenocarcinoma) cohort. Following the discovery of a number of technical problems that led to the over-irradiation, patients were distributed on three cohorts^23,24^. Cohort 1: Between 2004 and 2005, 24 prostate cancer patients were over-irradiated more than 20%, because of the improper use of a treatment planning system. For prescribed doses between 69 and 78 Gy, patients received doses between 81 and 120 Gy. Cohort 2: Between 2000 and 2006, 409 prostate cancer patients received 9–10% radiation overdose because of failure to consider doses delivered by daily portal imaging. For prescribed doses between 70 and 78 Gy, patients received doses between 76.7 and 85.5 Gy. Cohort 3: Between 1987 and 2000, at least 5000 patients were irradiated with 3% (1100 patients), 5.5% (3600 patients), or 7.1% (306 patients) more radiation than planned, depending on the photon energy used for their radiotherapy. The cause was an error in the homemade informatic program written to carry out calculations of monitor units (MU).

The present study investigated 208 patients with detailed characteristics reported in Table 1. According to their RB grade, determined by CTCAE method, at the time of blood sampling^25^, patients were divided into 4 severity grades (0, 1, 2 and 3/4) which included 84, 91, 25 and 8 patients respectively. Each patient with grade 2 or greater toxicity after RT was assessed by colonoscopy at the time of blood sampling, or during the follow-up. The CTCAE classification does not include grade 0 but in our study, the grade 0 patients included all patients who received an overdose of radiation but did not develop any symptoms at the time of blood sample collection (4.5 ± 2 years following end of RT, see Fig. 1). Blood samples were collected in 0.129 M sodium citrate tube and all patients provided informed consent. Several comorbidity factors that may influence the level of circulating vesicles, and other characteristics of the study participants are shown in Table 1. RB grade was assessed again during a median follow-up period of 4.8 years [3.4–6.3]^24^.

**Table 1:** Patient characteristics.

	Grade 0	Grade 1	Grade 2	Grade 3/4	*p*-value
N (toatal = 208)	84	91	25	8	
Included in cohort 1 (total = 5)	1	3	1	0	
Included in cohort 2 (total = 191)	76	85	23	7	
Included in cohort 3 (total = 12)	7	3	1	1	
Age (median)	74	74	75	76	0.81
Months between end of radiotherapy and blood collection (median)	62	54	58	44	0.33
Current smoker	44%	42%	52%	50%	0.92
Radical prostatectomy	18%	29%	12%	13%	0.30
Cardiovascular pathology	24%	21%	28%	25%	0.86
Anti-coagulant or antiplatelt drug (generated)	25%	26%	12%	38%	0.03
Anitcoagulant (AVK, Heparin)	7%	8%	8%	38%	0.03
Antiplatelet drug (aspirine, ticlid)	20%	20%	4%	12%	0.22
Hemoglobin at blood sampling (g/dL)	14.6 ± 1.61	14.4 ± 1.47	14.25 ± 1.35	15.00 ± 1.97	0.94

Subcohort dosimetry	Grade 0	Grade 1	Grade 2 or more	*p*-value	
N (total = 36)	10	17	9		
Mean dose to the prostate (Gy)	78.40 ± 4.65	77.75 ± 4.07	79.08 ± 4.14		
**Bladder dose quartiles (Gy)**					
Q75%	62.30 ± 10.59	68.49 ± 6.04	63.20 ± 13.56	0.36	
Q50%	34.82 ± 18.74	39.64 ± 13.57	40.61 ± 20.41	0.74	
Q25%	21.41 ± 18.21	16.59 ± 11.66	22.50 ± 15.15	0.94	
**Rectum dose quartiles (Gy)**					
Q75%	50.41 ± 20.76	67.57 ± 10.77	67.85 ± 8.57	0.02	
Q50%	30.25 ± 21.67	50.64 ± 20.23	51.46 ± 18.11	0.04	
Q25%	14.20 ± 9.20	29.80 ± 17.85	30.03 ± 19.42	0.06	
**Anterior rectal wall dose quartiles (gy)**					
Q75%	75.92 ± 2.43	75.10 ± 2.52	75.40 ± 2.12	0.61	
Q50%	70.94 ± 5.30	72.55 ± 3.11	73.24 ± 2.85	0.67	
Q25%	60.24 ± 9.84	62.55 ± 8.29	66.62 ± 6.41	0.32	

### MV isolation, labeling and flow cytometry analysis

Platelet-free plasma was obtained after centrifugation of blood sample at 450 g for 15 min at room temperature, followed by another centrifugation at 13,500 g for 5 min, 4 °C. The supernatant was distributed into 1.5 mL tubes and stored at − 80 °C. MVs were isolated from those samples by centrifugation at 20,600 g for 45 min, 4 °C.

MVs were labeled with Annexin V–FITC (IgG1), CD14–PE (clone RMO52), CD31–PE (1F11), CD41–PC5 (P2), CD3–PE (UC HT1) and CD235a–PC7 (KC16) and their corresponding isotype controls (Beckman Coulter, Villepinte, France). Incubation was carried out for 30 min in the dark at room temperature and MVs were resuspended in Annexin V Binding Buffer 1X (Beckman Coulter). To determine the absolute number of MVs per microliter in samples, 30μL of counting beads with an established concentration (Flow Count™ Fluorospheres, Beckman Coulter) were added to each sample.

Analyses were performed on a Cytomics FC500 flow cytometer (Beckman Coulter). MV detection was performed as previously described^26^. The cytometer was calibrated using a mix of fluorescent beads (Biocytex, Marseille, France) of different diameters to determine a detection window. The 3-μm beads indicated where the Flowcount beads appeared while the 0.5 and 0.9 μm beads determined the lower and upper detection limits of MVs, respectively. Only events included within this gate were further analyzed.

### Exosomes analysis

Size distribution and concentration of exosomes were determined using a NanoSight NS300 instrument (Malvern Panalytical, Malvern, UK). Vesicles in platelet free plasma were visualized and tracked by laser light scattering after a 1:100 dilution. Samples were analyzed with NTA 2.3 software (Malvern Panalytical).

### Statistical analysis

EV counts were expressed as median and range (25th percentile and 75^th^ percentile). Graphs represent the distribution of the populations with median (horizontal bar), 25^th^ and 75^th^ percentile (boxes), and 10th and 90th percentile (error bar). Analyses were performed by one-way ANOVA followed by Tukey’s multiple comparison test using GraphPad Prism software v.5.03 (GraphPad Software, San Diego, CA). When data did not show a Gaussian distribution, Kruskall-Wallis test was used followed by Dunn’s multiple comparison test. Results with a *P*-value < 0.05 were considered significant.

### Electronic microscopy

Pelleted MVs were fixed with a 2.5% glutaraldehyde solution for 1 h at 4 °C. After a centrifugation at 20,000 g for 120 min at 4 °C the solution was replaced by PBS. The pellet was solidified by addition of agarose before fixation with osmium tetroxide 2%.

### Cell culture

The human U937 monocytic leukemia cell line (ATCC) was cultured with RPMI 1640 supplemented with FBS 10%, Hepes and Penicillin/Streptomycin (50 IU/ml and 50 μg/ml, respectively) (Life Technologies, Villebon sur Yvette, France). Primary human microvascular endothelial cells (HMVEC) and lymphatic endothelial cells (HLMVEC, Lonza Verviers, Belgium) were cultured with EBM-2 medium supplemented with hEGF (0,1%), hydrocortisone (0,04%); GA-1000 (0,1%), FBS (20%), VEGF (0,1%), hFGF-b (0,4%), R3-IGF-1 (0,1%) and ascorbic acid (0,1%) (Lonza). After thawing, all cells were cultured in 75-cm^2^ flasks for amplification, then in 6-well plates by using Trypsin–EDTA (1X) 0.05% (Life Technologies). After reaching confluence state, cells were irradiated using a ^137^Cs source at 10 Gy, 20 Gy and 40 Gy for all cell types.

### MV-dependent functional assays

The MV-dependent TF activity was measured using a procoagulant activity assay, as described earlier^27^. The MV-dependent plasmin generation assay was conducted using a chromogenic test, as previously described^28^.

### MV proteomics analysis

Protein extraction, tandem mass tagging labeling, LC–MS/MS analysis, assignment of MS/MS spectra, and quantitative data analysis were performed as previously described, starting from 50 µg MV lysates^29^. Statistical analysis of radiation induced protein changes was conducted using the LIMMA package of R framework and Benjamini–Hochberg correction for multiple comparisons^30^.

### Association between organ irradiation and EV secretion

The association between the organ dose distribution and MV counts, or with exosome enumeration, was investigated through a scalar on function regression^31,32^. In this approach, the dose distribution is represented by the quantile function which achieves a “synchronization” between the irradiation profiles in order to exploit the patient-to-patient irradiation heterogeneity in the analysis^33^.

The general formulation of a scalar on function regression model is given by:

$$ {\text{EV secretion }} = \, \int {\beta {\text{Dose}}\left( {\text{x}} \right) \, \times {\text{ QDose}}\left( {\text{x}} \right)} {\text{ dx }} + Covariates$$

where QDose(x) is the quantile function predictor and βDose(x) is the functional parameter of the model providing an exposure weighting over the whole range of dose values. A positive (negative) value of the functional parameter βDose(x) can be interpreted as an increase (decrease) of EV secretion when the organ is exposed to the corresponding quantile doses. Note that additional confounding factors can be included in the model (age, diseases, concomitant treatments, etc.). The functional parameter was estimated using penalized spline functions using the REFUND Package of R software^30^.

The reconstruction of the real dose distribution to the rectum, anterior rectal wall and bladder was possible only for a subcohort of 36 patients from the cohort 2 due to the overwriting procedure of the dosimetric records occurring in the hospital at this time. The relative dose volume histograms (DVHs) were calculated in 0.03 Gy dose bins, using treatment planning systems Eclipse and CadPlan from Varian Medical Systems (Palo Alto, CA).

### Association between EV secretion and RB grade during the post blood sampling follow-up

The association between the EVs and a worst RB grade during the post blood sampling follow-up was investigated using a multivariate logistic regression (Table 2). In this analysis, an event consists in developing a more severe RB during the post blood sampling period compared to the RB grade scored at the time of blood sampling. The model was adjusted on age, prescribed radiation dose, the delay between the radiotherapy and the blood sampling as well as the toxicity grade at the time of blood sampling. Adverse treatment such as anti-aggregates and anti-hypertensives, laser treatment, transfusion of packed cells, and particularly anticoagulants (*p* = 0.03, Table 1) were tested as confounding factors and model selection was performed using the Akaike information criteria^34^.

**Table 2:** Association between cell derived MVs or exosomes with a worst RB grade during the post blood sampling follow-up, according to logistic model.

	Estimate	SE	95% CI	OR	OR 95% CI	*p*-value		
log(PMVs)	0,376	0,190	− 0,004	0,756	1,456	0,996	2,129	0,048
Log(MMVs/EMVs)	1,128	0,362	0,404	1,853	3,089	1,497	6,379	0,002

All the analyses were adjusted on age, prescribed radiation dose, adverse treatment, the delay between the radiotherapy and the blood sampling as well as the toxicity grade at the blood sampling.

MV and exosome data were incorporated in the model both in a quantitative (using a log-transformation) and compositional manner. Based on the cellular origin of MVs obtained by flow cytometry analysis, the MV composition of each patient was summarized by a four-component vector, listing the proportion of MVs derived from platelets, endothelial cells, monocytes and others. These compositional data were then treated using the Aitchison geometry, as described^35^. In the present study, we adopted an isometric log-ratio transformation for an isometric mapping from the simplex sample space to the real space^36^.

## Results

### MV plasma level in patients and cellular origin

**Figure 2:**
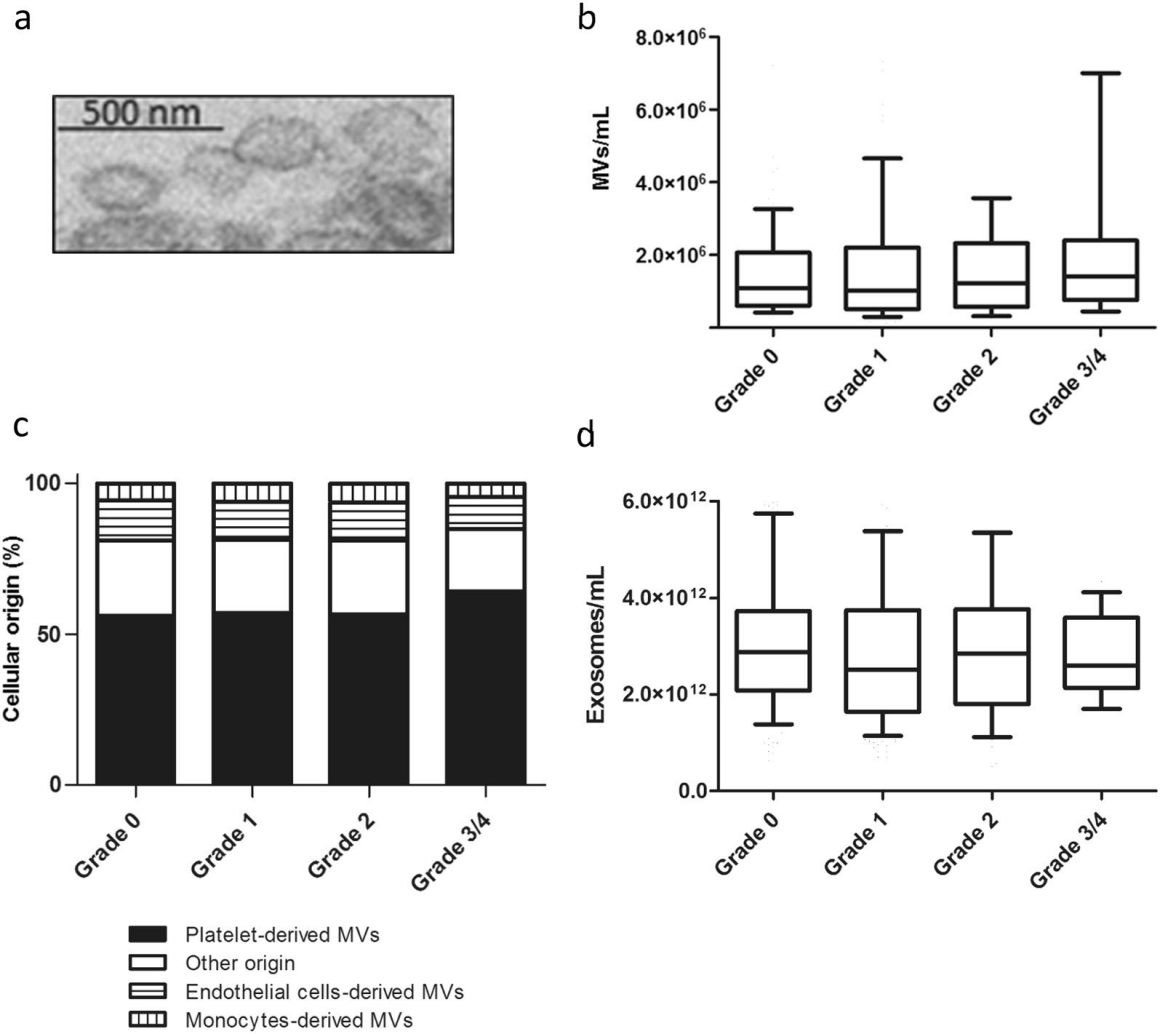
Circulating EV characterization in patients. (**a**) Morphology of circulating EVs isolated from grade 0 patients by transmission electron microscopy. Scale bars: 500 nm. (**b**) Levels of circulating MVs in patients, according to their RB grade. MVs were enumerated after labeling with Annexin V using flow cytometry. (**c**) Cellular origin of all enumerated MVs. Data are expressed as percentage of MPs enumerated in (**b**). (**d**) Levels of circulating exosomes. Exosomes were enumerated using NTA technology with Nanosight. Data information: In (**b**, **d**) data are presented as median (horizontal bar), 25th and 75th percentile (boxes), and 10th and 90th percentile (error bar). EV levels were analyzed by ANOVA followed by Dunn’s multiple comparison test. Results with a *P*-value < 0.05 were considered significant.

EVs were visualized by electron microscopy after being isolated from over-exposed patients’ blood, confirming expected size range (Fig. 2a). The number of Annexin V positive MVs was similar between the different grades of severity but tended to be increased in the highest grade of severity compared to the lower grades (Fig. 2b).

We observed that MVs were mainly derived from platelets (PMVs) and in a less proportion, from endothelial cells (EMVs) and monocytes (MMVs) (Fig. 2c). In addition, we noticed a significant increase in the proportion of PMVs in patients with grade 3/4 compared to grades 0, 1, and 2 (*P* < 0.05), with coincident reduced proportion of EMVs and MMVs. This observation likely reflects that different cell types are activated or undergo apoptosis in patients with high severity grade compared to lower grades. The increase of PMV numbers may be related to the clinical symptoms associated to this grade of severity, including mainly dysregulation of the hemostatic process.

Surprisingly, we observed no significant modification of the amount of circulating exosomes between all the different grades of severity (Fig. 2d).

### Association with more severe complication after radiotherapy

**Figure 3:**
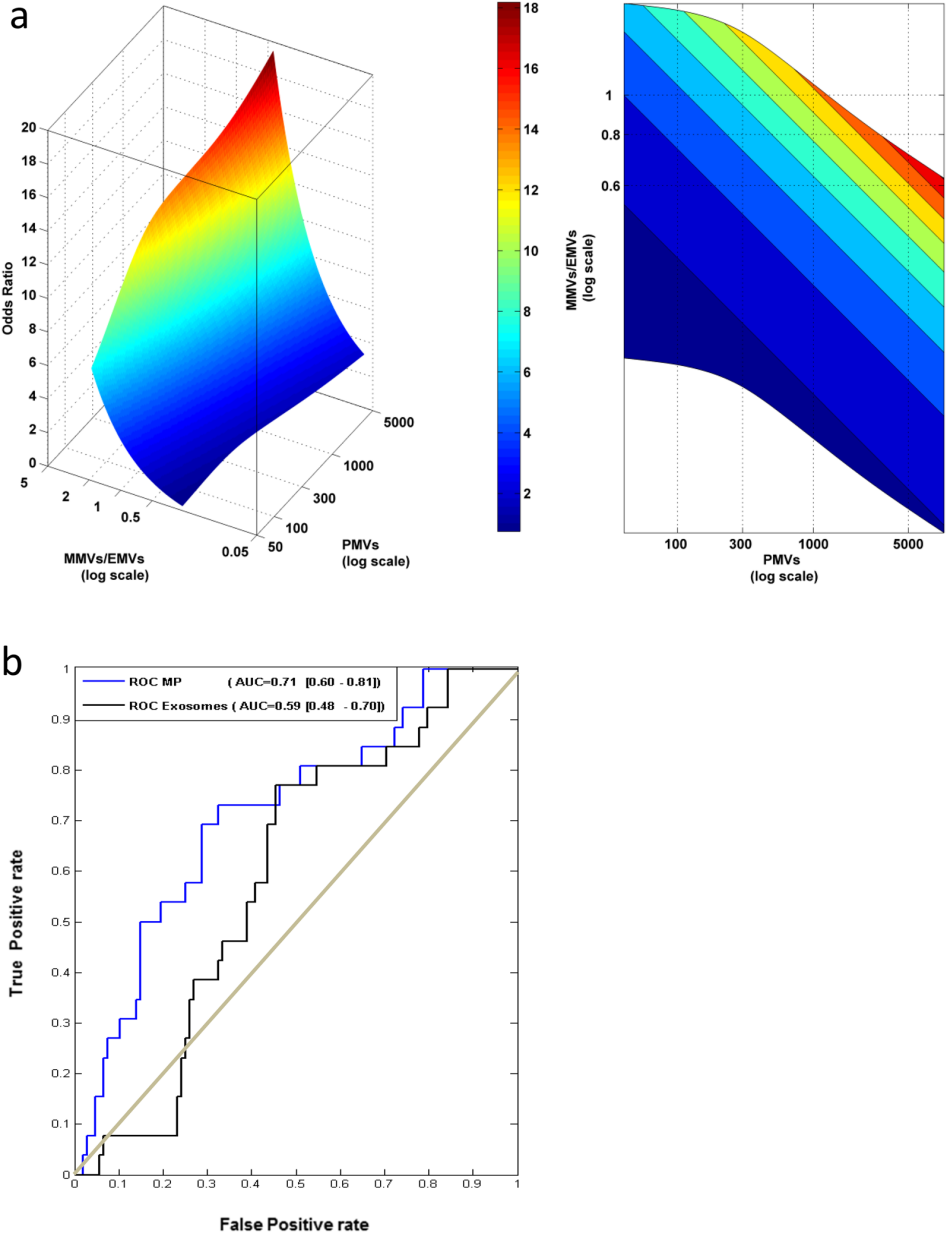
Risk prediction model for RB grade worsening. (**a**) Odds-ratio of the logistic model based on MVs illustrated as a bivariate function of PMVs, EMVs and MMVs. Left: 3D color map surface; right: contour projection in the XY plane. (**b**) Receiver operating characteristic (ROC) for the logistic model based on MVs data (blue) or exosomes (black) and their respective area under the curve (AUC) with 95% confidence intervals between brackets.

Multivariate logistic regression analysis revealed a significant association between PMV, MMV and EMV levels at the time of blood sampling, with the occurrence of a worst RB grade during the patient’s follow-up. Table 2 shows that an increased number of PMVs, and an increased number of MMVs relative to the number of EMVs (MMVs/EMVs ratio), were associated with higher grade of RB severity during follow-up, compared to the toxicity grade at the time of blood sampling (odds ratio (OR) = 1.45, CI [1.00–2.13], and OR = 3.09, CI [1.50–6.38] respectively). For example, an increase in the PMV count by 20% enhances by 7% (95% CI: [0.03%, 14.6%]) the risk to have a higher RB grade compared to the grade scored at the time of blood sampling. The analysis also shows that an increase of 20% of the MMVs/EMVs ratio increases by 23% (95% CI: [8%; 39%]) the risk of a more severe RB grade compared to the grade scored at the time of blood sampling. An illustration of the MV quantitative (PMVs) and compositional (MMVs/EMVs ratio) conjugate effects on the OR is illustrated in Fig. 3a.

In order to determine whether exosome counts could be relevant for toxicity worsening risk estimation, receiver operating characteristic (ROC) for the logistic model was computed based on MV data vs exosomes (Fig. 3b), showing a higher discrimination ability for the former over the latter model (AUC = 0.71, CI [0.6;0.81] vs AUC = 0.59, CI [0.48;0.7]). This result indicates in particular that exosome counts bring no significant information for patient classification since the confidence interval of its AUC include 0.5 (Fig. 3b).

### Proteome characterization of MVs

**Figure 4:**
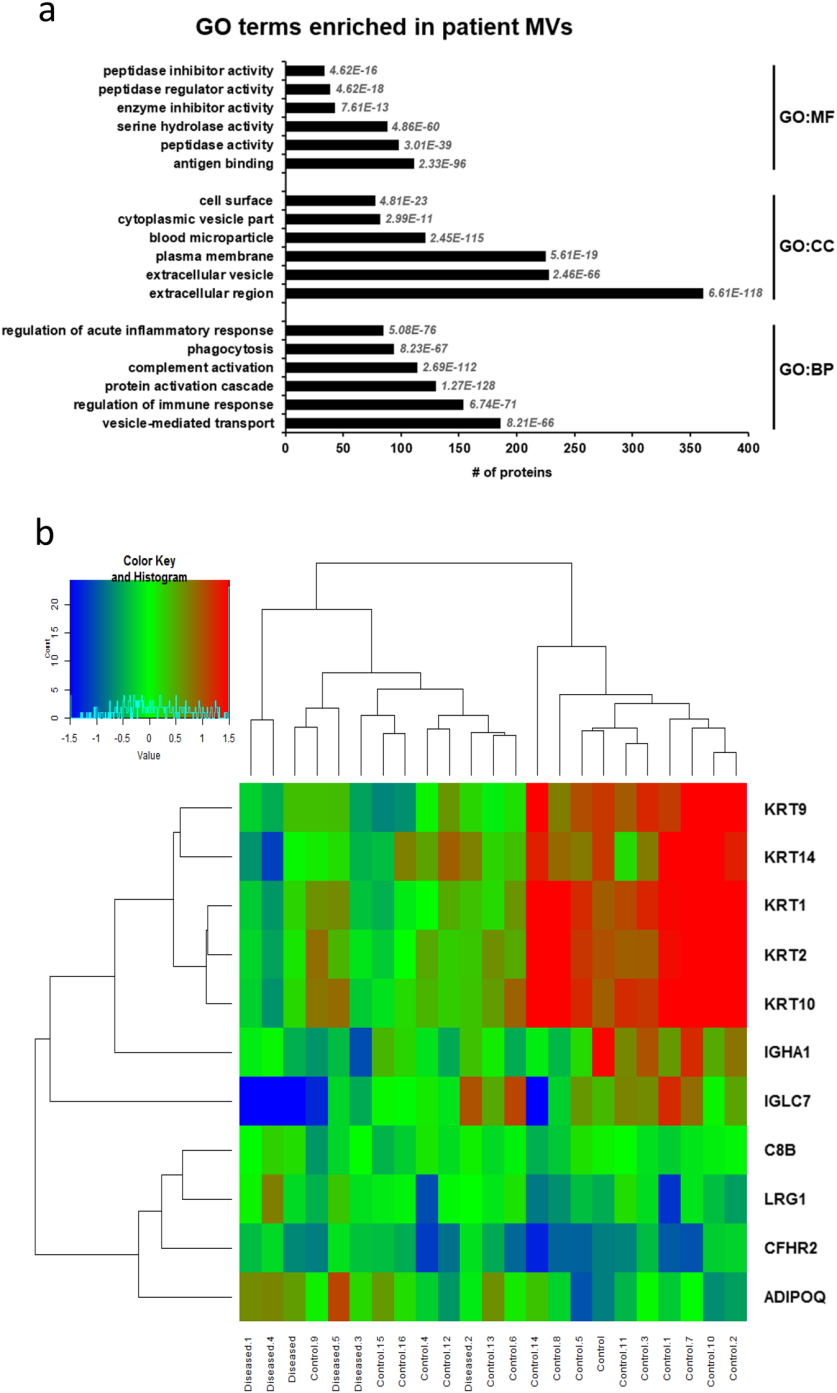
Proteomics characterization of patient MVs. (**a**) Functional categorization of enriched proteins in circulating MVs, based on gene ontology (GO) annotations. FDR adjusted P-value of enrichment is indicated in italics for each protein category. (**b**) Heat map representation of MV protein profiles in grade 0 vs grade ≥ 2 patients (for proteins with *P* < 0.05 after LIMMA test and FDR < 0.1). The row displays proteins and the column represents the samples, harvested from patients with RB grade ≥ 2 (named Diseased) or with grade 0 (named Control). Proteins significantly decreased are displayed in blue, while proteins significantly increased are displayed in red. The heatmap was generated using the “heatmap” command available in the Limma Package (version 3.50) of the R software (version 4.1, URL : https://www.r-project.org/).

Liquid chromatography (LC)-mass spectrometry (MS)/MS was used to determine the protein content of MVs in a subset of patients across all toxicity grades (see Supplementary Table S1 for the complete list of detected protein IDs and quantification results). First, we performed global Gene Ontology (GO) term enrichment analysis to obtain a general overview of enriched functional classes within the ~ 500 MV proteins identified by LC–MS/MS (Fig. 4a). Representative categories of the most significantly enriched GO terms within the molecular function category (GO:MF) were related to enzyme function associated to coagulation and inflammatory system. In the cellular component category (GO:CC), the top terms were linked to “extracellular region”, “plasma membrane”, and “blood microparticles”, highlighting the importance of both biogenesis and extracellular localization of vesicles. Interestingly, in the biological process category (GO:BP), the top terms were involved in complement activation, regulation of inflammatory response, coagulation and wound healing process. These observations underscored the importance of EVs in the pathophysiology associated with the coagulation and inflammatory response, characteristic of the degree of severity of radiotherapy-induced late toxicity in abdominopelvic cancer.

Next, an unsupervised, hierarchical clustering analysis identified a set of MV proteins with differential abundance between patients with grade 0 and patients with grade ≥ 2, with a false discovery rate set to < 0.1 (Fig. 4b). Proteins known to have a beneficial effect on angiogenesis, vesicle or lipid transport, were found overrepresented in MVs from patients with severity grade ≥ 2 compared to grade 0, including ADIPOQ, CFHR2 and C8B. Additionally, reduced abundance of the keratin protein family members KRT-1, 2, 9, 10 & 14, associated with structural filament organization, was observed in MVs from patients with toxicity grade ≥ 2 compared to grade 0.

### Correlation between the irradiation dose/volume to the organ and MVs

**Figure 5:**
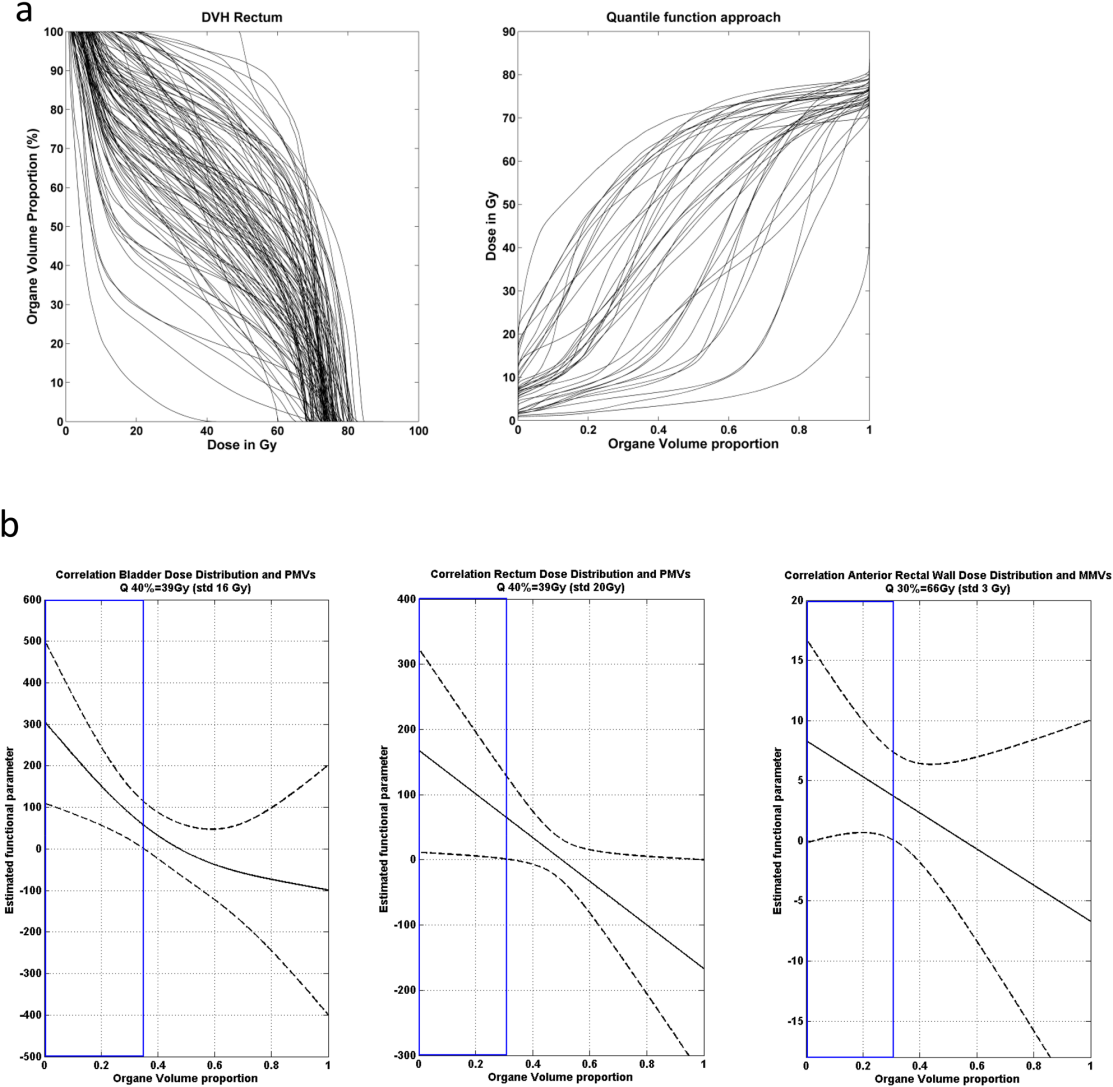
Correlations between DVHs and MVs. (**a**) Examples of patient-to-patient variability of rectum exposure through dose distribution histograms (DVH, left panel) and quantile functions (right panel). (**b**) Estimated functional parameters of the scalar on function regression representing the association of organ exposure with MV enumeration. Dashed lines represent the corresponding 95% confidence bands. The blue panels indicate a significant association between quantile doses and the number of MVs.

The dose–response relationship between the organ dose distribution and EV secretion was investigated through a scalar on function regression, taking advantage of the dose/volume histogram (DVH) information for anterior rectal wall, bladder, and rectum available for a subset of 36 patients (Fig. 5a). This analysis revealed a positive correlation between the number of MMVs and the doses received by the anterior rectal wall until D30% (66 Gy, std 3 Gy) (Fig. 5b, right panel). Additionally, a positive correlation was found between the rate of PMVs and the doses received by the bladder until D40% (39 Gy, std 16 Gy), and by the rectum until D40% (39 Gy, std 20 Gy) (Fig. 5b, left and middle panels). More importantly, no correlation was found with the most elevated doses, indicating that the number of secreted MVs was mainly associated with the volume of organ exposed to the range of low to moderate doses, rather than hot spots of highest doses. Surprisingly, no correlation was found between exosome counts and organ dose distribution, suggesting that MVs were the only EV population modulated by radiation exposure in this cohort.

### Effect of irradiation on MV functional activity

To provide further insight into the functional consequences of irradiation on MVs, a series of in vitro coagulation assays was conducted on EMVs and MMVs. MV procoagulant function was investigated by measuring tissue factor (TF) activity of MVs produced by endothelial cells and monocytes following exposure to different radiation doses. Our results indicate a trend toward a dose- and time-dependent increase of MV-related TF activity after irradiation (Supplementary Fig. S1a–c), with highest effect displayed by MMVs (Supplementary Fig. S1a). TF activity of MMVs increased in a dose-dependent manner from 3 h until 1 day after radiation exposure, while TF activity of EMVs peaked at 6 h after irradiation and then tended to decrease from day 1 onward (Supplementary Fig. S1a–c).

A plasmin generation assay was used to analyze the fibrinolytic activity of EMVs and MMVs after irradiation. Only MMVs were found to induce plasmin generation, in irradiated and non-irradiated conditions (Supplementary Fig. S1d). The plasmin generation was detected from 3 h after exposure of monocytes to 40 Gy irradiation and from 6 h after 20 Gy irradiation (but not 10 Gy). In non-irradiated conditions, plasmin generation induced by MMVs was observed at 1 day. After 3 days of culture, irradiation affected the level of plasmin generated by MMVs (Supplementary Fig. S1d).

## Discussion

The discovery of a valid and reproducible biomarker for the complications of radiotherapy is still a major clinical challenge. The present study demonstrated that the number of circulating EVs tended to be increased in patients with the highest grade of toxicity following accidental abdominopelvic complications. More importantly, we found that the proportion of MMVs relative to EMVs (MMVs/EMVs ra), as well as the number of PMVs, were associated with a higher RB grade during follow-up, compared to the toxicity grade at the time of blood sampling. Finally, MV studies provided important information on cellular pathology and identified protein signatures associated with radiation exposure and clinical complications.

EVs tended to be increased in patients with grade 3/4 compared to grade 0, 1 and 2 without reaching statistical significance. However, there is a possibility of MV number variation earlier after radiotherapy. In addition, MVs were mainly derived from platelets and in a less proportion from endothelial cells and monocytes. Strong evidence suggests that MVs from endothelial, platelet, and leukocyte (LMVs) origin may characterize and participate to endothelial dysfunction^37^.

Similarly, patients with hypertension have been shown to have elevated PMVs, EMVs and MMVs as compared to normotensive patients^38^. Different studies have shown a raise in EMVs and PMVs in patients with acute coronary syndrome^39,40^. These studies show MVs as promising markers of cardiovascular diseases. Based on our findings, MVs could also discriminate patients with distinct severity grades of chronic radiation late effects.

Additionally, EMVs and MMVs have been considered as favorable surrogate markers for reflecting the degree of endothelial cell dysfunction. MMVs could be considered as pro-inflammatory vesicles that increase vascular inflammation^41,42^. Inflammation stimulates EV release which could initiate a positive feedback loop through their capacity to stimulate effects on adhesion molecule expression, cell–cell interaction, or cytokine release^9^. Therefore, the combination of EMV, MMV and PMV counts could be more reflective of the endothelial health than each MV sub-population individually.

Furthermore these MVs could be implicated in a cross-talk communication to promote the initiation and the expansion of the coagulation process^43,44^. EMVs and MMVs are known to be procoagulant due to the presence of TF and phosphatidylserine at their surface^45,46^. More specifically, several studies suggested that MMVs could constitute the most important source of circulating TF and are able to transfer their TF to the activated platelets to propagate the coagulation process^43,47^. Thereby, MMVs could play a central role in the cross-talk between inflammation and thrombosis. However, EMVs and MMVs can also harbor some anti-coagulant proteins on their membranes, suggesting that they can display a full spectrum of regulator of the hemostatic process^48^ and our results confirmed that MMVs could exert both pro- and anti-coagulant activity in vitro.

Interestingly, the MMVs/EMVs ratio was associated with higher rectal hemorrhage, suggesting that MMVs are implicated in the initiation/propagation of deleterious rectal bleeding. In this regard, a previous study demonstrated that MMVs can induce endothelial cell thrombogenicity and apoptosis. This could impair endothelium and aggravate bleedings^49^. More importantly, the risk estimate increased with the PMP implementation, suggesting that PMPs also contribute to the rectal bleeding state. In sharp contrast, no correlation was established between exosomes and RB grade aggravation.

Proteomic analysis of PMVs, EMVs and LMVs has provided significant insight into MV composition and biological activity^50^. In our study, we have shown that the proteomic analysis of MVs from a subset of patients with no toxicity vs highest toxicity grades resulted in the clustering of all grade ≥ 2 patients, thereby providing insight towards a protein signature of circulating EVs for highest toxicity grades. Among the proteins overrepresented in MVs of grade ≥ 2 patients were adiponectin and leucine-rich alpha-2 glycoprotein (LRG1), which have been shown to promote angiogenesis and exert anti-inflammatory activity. These proteins have been proposed as putative biomarkers for several malignancies, including prostate, ovarian and pancreatic cancer^51–53^. Interestingly, elevated serum levels of both adiponectin and LRG1 have been found in patients with ulcerative colitis, an inflammatory bowel disease usually affecting the colon and rectum^54,55^. Additionally, increased abundance of proteins involved in alternative complement pathway regulation (CFHR2, C8B), were also identified in MVs of grade ≥ 2 patients.

Our retrospective study design allows examining not only variations of different biological parameters with time progression, but also with severity grade. Protein composition of MVs could allow the identification of most severe toxicity grades. EVs may present advantage of a non-invasive approach to evaluate complication after radiotherapy.

Interestingly, we found a positive correlation between MMV counts and the doses received by the anterior rectal wall. This was in accordance with studies demonstrating the long-term presence of inflammatory cells and collagen depositions under the rectal wall in patients with chronic radiation proctitis^56^. Additionally, we obtained a positive correlation between PMV counts and the doses received by the bladder and the rectum. Similarly, it has been shown that platelet derived growth factor (PDGF) expression was upregulated in bladder after irradiation, and possibly involved in fibrous contracted bladder following pelvic irradiation and in irradiation-induced vascular changes^57^. Interestingly, our results suggest that MV secretion is essentially dependent on the volume of organ irradiated with the range of low to moderate doses, which represents a large volume of tissue, and therefore a large proportion of MV-secreting vascular cells.

Our study conducted on a cohort of 208 patients overexposed to ionizing radiations suggests that EVs can be used as biomarkers for diagnosis of complication of RT. Additionally, patients display specific EV protein profiles according to their radiation-induced toxicity grade. Our study provides insights into the characterization of EVs in pathological conditions.

However, some limits of our retrospective study need to be highlighted, insofar as results were obtained on a selective group of patients, with analyses performed several years after the end of radiotherapy. Thus, the main difference with a medical designed cohort is mainly the baseline status of the recruited patients. Indeed, at the moment of blood sampling, numerous patients had already developed Grade 1 or greater rectal bleeding and our results focused on the role of MV to the prognostic of a worsening grade post blood sampling.

To address the role of MV as a predictive tool in standard RT context, we conducted an European multicentric prospective study (MEDIRAD: Implications of Medical Low Dose Radiation Exposure; Grant Agreement: 755523, clinical trial NCT03297346) for the evaluation of EVs in a cohort of breast cancer radiotherapy patients at different time points, including before, at the end of the RT and 6 and 24 months after RT. It will provide information on the evolution of biomarkers associated with clinical symptoms^58^. More importantly, circulating EV biomarkers could allow for early detection of deleterious radiation effects and may be a support for therapeutic strategy. Indeed, previous studies have described the potential use of MVs for risk estimation in various cardiovascular diseases. In particular, an association was shown between the Framingham risk score, which is used as a prediction tool for cardiovascular disease risk, and plasma concentrations of PMVs, EMVs and LMVs^59,60^. Moreover, an increase of MV levels was shown to be predictive of cardiovascular death and acute coronary syndromes, and could participate to the risk stratification in stable coronary artery disease patients^59,60^.

More investigation will allow to consider the EVs as non-invasive circulating biomarker allowing the identification of over-irradiated patients and risk evaluation of radiotherapy complication.

## Supplementary Information

Supplementary Information 1.

Supplementary Information 2.

Supplementary Information 3.

## Data Availability

All data generated and analyzed during this study are included in this published article (and its supplementary information files).
