# Supplementary material for: Circulating microvesicles correlate with radiation proctitis complication after radiotherapy
Source: Sci Rep. 2023 Feb 4;13:2033. doi: 10.1038/s41598-022-21726-y (PMC9899237; doi:10.1038/s41598-022-21726-y)
Supplement: Supplementary file 1 — Supplementary Information 1. [file 41598_2022_21726_MOESM1_ESM.docx]

**Circulating microvesicles correlate with radiation proctitis complication after radiotherapy**

**Supplementary data**

**Supplementary materials and methods**

**MV-dependent Tissue Factor activity assay**

The MV-dependent tissue factor (TF) activity was measured using a procoagulant activity assay adapted for high sensitivity from Lee *et al.* [*^1^*](#_ENREF_1) MVs were pelleted from 1 mL of cell supernatant by centrifugation at 24 000 g for 60 min at room temperature, washed in HEPES buffer (150 mM NaCl, 20 mM HEPES and 0.1% NaN3, pH 7.4) and resuspended in 145 μL of HEPES. 70 μL were pre-incubated for 30 min at 37°C with either an inhibitory anti-TF monoclonal antibody (10 μg/ml final, clone SBTF1, BioCytex) or a control antibody (10 μg/ml, clone a-DNP 2H11-2H12, BioCytex). Then, 8 μL of HEPES-Ca²+ buffer containing coagulation factor (F) VII and FX (Enzyme Research Laboratories, Stago, Lille, France) was added to each 70 μL sample, to produce final concentrations of 10 nM, 190 nM and 5 mM CaCl2, respectively and incubated for another 2 h at 37°C. Generation of FXa was blocked by the addition of 10 μL of EDTA buffer (10 mM HEPES and 200 mM EDTA) and a proprietary FXa fluorogenic substrate (1 mM final, kind gift from BioCytex). Finally, the fluorescence at 390 nm excitation/460 nm emission was measured for 15 min at 37°C on a microplate fluorescence reader (Fluoroskan, CAT instrument, Stago). Maximum reaction velocity (Vmax) was calculated with the associated software (Ascent Software, Luqa, Malta) using 5 points and used as a measure of the generated FXa. Vmax were corrected by subtracting those generated in the presence of SBTF1 antibody from those generated in the presence of the control antibody. Data from plasma-purified MVs were expressed as TF (fmol) by comparison to a calibration curve generated using recombinant TF (6 pmol initial concentration).

**MV-dependent plasmin generation assay**

The capacity of MVs to activate plasminogen was determined by incubating plasminogen at a fixed concentration of 4 μM (Diagnostica Stago, Asnières, France) with the purified MVs overnight at 37°C. Then, a chromogenic substrate selective for plasmin ([methyl-malonyl]-hydroxypropylarginine-p-nitroanilide, CBS 0065, Diagnostica Stago) was added at a final concentration of 2 mM. The chromogenic substrate was cleaved by the plasmin, which had formed from the plasminogen with the help of MVs. The released p-nitroaniline was detected by measuring its absorbance at 405 nm as a function of time. The kinetics of plasmin generation were followed for 2 h at 37°C, and the Vmax was measured in the linear part of each curve. The results were expressed in units of A405nm×10-3/min.

**MV proteomics analysis**

*Protein Extraction and Digestion*

Each MV pellet was homogenized in lysis buffer (8M Urea in 50mM Ammonium Bicarbonate and protease inhibitor cocktail) and protein concentration was measured by Qubit Protein Quantification Assay (Thermo Fisher, Rockford, IL). An equal amount of protein lysate from each sample was pooled as a common reference to generate ratios in the labeling experiment. MV lysates (50 μg) were reduced with 10 mM tris(2-carboxyethyl)phosphine (TCEP) and alkylated with 18.75 mM iodoacetamide in the dark for 30 min. Protein was extracted by methanol/chloroform precipitation. Pellets were dried, resuspended in 100 mM Triethylamonium bicarbonate (TEAB) according to the manufacturer’s protocol. Trypsin was added to the protein extract in a 1:40 enzyme to protein ratio, and incubated for 16 hours at 37°C. Next day, samples were centrifuged for 30 min at 14000 rpm to remove insoluble materials, and 2.5 μg of the peptide mixture was used for peptide quantification assay (Thermo Fisher).

*Tandem Mass Tagging Labeling*

Isobaric labeling of digested MV lysates was performed using the 10-plex tandem mass tag (TMT) reagents (Thermo Fisher). TMT reagents (0.8 mg) were dissolved in 41 μl of dry acetonitrile (ACN), and 8.2 μl were added to 20 μg of digested lysate in 100 mM TEAB. After 1 hour incubation at room temperature, the reaction was quenched by adding 3 μl of 5% hydroxylamine. Labeled peptides were combined and dried for the subsequent high pH reverse phase peptide fractionation (Thermo Fisher). Eight fractions were generated for each combined set.

*LC-MS/MS Analysis*

Each TMT labeled fraction was reconstituted in a solution of 2% ACN, 2% formic acid for MS analysis. Peptides were eluted from the easy-spray column (50 cm) using a Dionex Ultimate 3000 Nano LC system with a 30-min gradient from 5% to 20% buffer B (100% ACN, 0.1% formic acid) and a 10-min gradient from 20% to 30% buffer B. The gradient was switched from 30% to 85% buffer B over 1 min and held constant for 3 min. Finally, the gradient was changed from 85% buffer B to 98% buffer A (0.1% formic acid in water) over 1 min, and then held constant at 98% buffer A for 20 min. The application of a 2.0 kV distal voltage electrosprayed the eluting peptides directly into the Thermo Fusion Tribrid mass spectrometer equipped with an EASY-Spray source (Thermo Fisher). Mass spectrometer-scanning functions and HPLC gradients were controlled by the Xcalibur data system (Thermo Finnigan, San Jose, CA).

For all experiments, the instrument was operated in the data-dependent mode. We collected Fourier transform-based (FT) MS1 spectra at a resolution of 120K with an automated gain control (AGC) 400,000, a max injection time of 50 ms. The 10 most intense ions were selected for MS/MS. Precursors were filtered according to charge state (z = 2-7), and monoisotopic peak assignment. Previously interrogated precursors were excluded using a dynamic exclusion duration of 60 s. For the FTMS3 method, ion trap (IT) MS2 spectra were collected at AGC of 100,000, max injection time of 105 ms, and collision-induced dissociation (CID) collision energy of 35%. FTMS3 spectra utilized the same Orbitrap parameters as FTMS2 method, except Higher-energy collisional dissociation (HCD) collision energy was increased to 55% to ensure maximal TMT report ion yield. Synchronous-precursor selection was enabled to include up to 3, 6, or 10 MS2 fragment ions in the FTMS3 scan.

*Database Search and Assignment of MS/MS Spectra*

Tandem mass spectra from raw files were searched against a human protein database using the Proteome Discoverer 2.1 (Thermo Finnigan). The Proteome Discoverer application extracts relevant MS/MS spectra from the raw file and determines the precursor charge state and the quality of the fragmentation spectrum. The Proteome Discoverer probability-based scoring system rates the relevance of the best matches found by the SEQUEST algorithm. The human protein database was downloaded as FASTA-formatted sequences from UniProt protein database (released in October, 2016). The peptide mass search tolerance was set to 10ppm. A minimum sequence length of 7 amino acid residues was required. Only fully tryptic peptides were considered. To calculate confidence levels and false positive rates (FDR), Proteome Discoverer generates a decoy database containing reverse sequences of the non-decoy protein database and performs the search against this concatenated database (non-decoy + decoy).

*Determination of TMT Reporter Ion Intensities and Quantitative Data Analysis*

For quantification, a 0.003 m/z (10plex TMT) window centered on the theoretical m/z value of each reporter ion was queried for the nearest signal intensity. Reporter ion intensities were adjusted for the isotopic impurities of each TMT reagent according to the manufacturer’s specifications. The signal-to-noise (S/N) values for all peptides were summed within each TMT channel, and each channel was scaled according to the interchannel difference of these sums to account for differences in sample handling. For each peptide, a total minimum sum S/N of 400 and an isolation purity greater than 75% was required. Statistical analysis of radiation induced protein changes was conducted using the linear model for microarray (LIMMA package of R)[^2^](#_ENREF_2) framework and Benjamini-Hochberg correction was applied for multiple comparisons.

**Supplementary Table and Figure legend**

**Supplementary Table S1: Complete list of detected protein IDs and quantification results from MV proteomic analysis**

**Supplementary** **Figure S1 – Effect of irradiation on MV functional activity**

(a-c) Tissue factor (TF) activity assay on MVs derived from human microvascular endothelial cells (a), lymphatic endothelial cells (b) and monocytes (c). TF activity was measured at 3h, 6h, 24h, or 72h after exposure to various doses of radiation.

(d) Plasmin generation assay on human monocyte-derived MVs. All MVs were isolated from culture medium. No histogram means no generation of plasmin.

**Supplementary references**

1 Lee, R. D. *et al.* Pre-analytical and analytical variables affecting the measurement of plasma-derived microparticle tissue factor activity. *Thrombosis research* **129**, 80-85, doi:10.1016/j.thromres.2011.06.004 (2012).

2 A language and environment for statistical computing (R Foundation for Statistical Computing, Vienna, Austria, 2014).
