# Supplementary figures and images for: Circulating microvesicles correlate with radiation proctitis complication after radiotherapy
Source: Sci Rep. 2023 Feb 4;13:2033. doi: 10.1038/s41598-022-21726-y (PMC9899237; doi:10.1038/s41598-022-21726-y)

## Slide 1
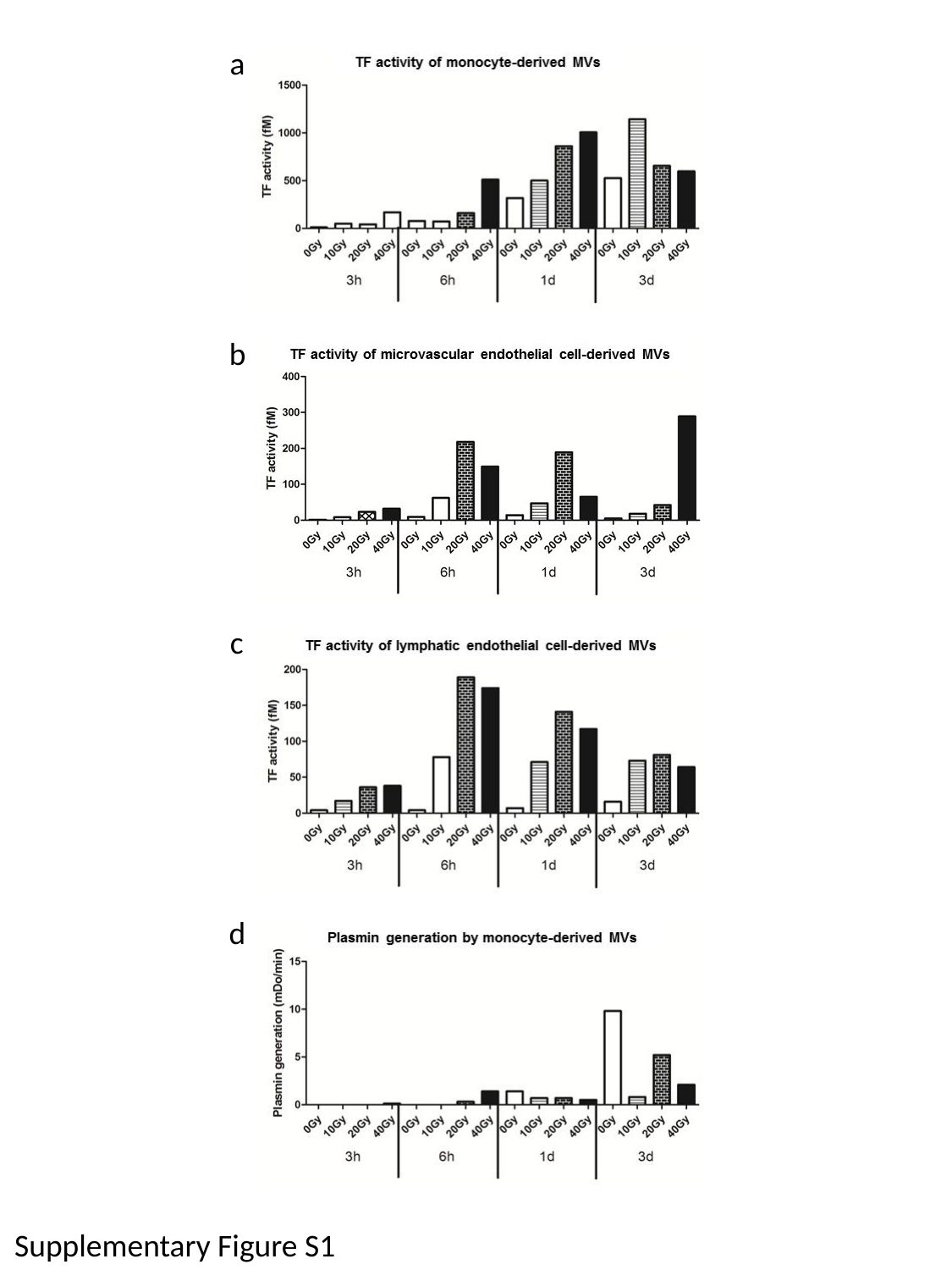

a
b
c
d
Supplementary Figure S1

Supplement: Supplementary file 2 — Supplementary Information 2. [file 41598_2022_21726_MOESM2_ESM.pptx]
